# Process Evaluation of a School‐Based Intervention Promoting Sleep Health in Adolescents: A Mixed‐Methods Study

**DOI:** 10.1111/josh.70061

**Published:** 2025-08-12

**Authors:** Maj‐Britt M. R. Inhulsen, Maartje M. van Stralen, Femke van Nassau, Vincent Busch

**Affiliations:** ^1^ Department of Health Sciences, Faculty of Science, Amsterdam Public Health Research Institute Vrije Universiteit Amsterdam Amsterdam the Netherlands; ^2^ Sarphati Amsterdam, Public Health Service (GGD) Amsterdam the Netherlands; ^3^ Department of Public and Occupational Health, Amsterdam Public Health Research Institute Amsterdam University Medical Centre, Vrije Universiteit Amsterdam the Netherlands

**Keywords:** adolescents, implementation, school‐based intervention, sleep

## Abstract

**Background:**

Poor sleep health is increasingly recognized as a public health issue. Despite the potential of school‐based interventions, few have successfully improved adolescent sleep health. To enhance intervention effectiveness, feasibility, and relevance, it is essential to understand barriers and facilitators affecting the adoption, implementation, and sustainment. This study evaluates these aspects for the sleep health promoting school‐based intervention Charge Your Brainzzz in Dutch secondary schools.

**Methods:**

We conducted 12 semistructured interviews with teachers from five implementation schools, complemented by 372 questionnaires completed by second‐ and third‐grade students from various educational tracks. Qualitative data were thematically analyzed and quantitative data were analyzed using descriptive statistics and tests.

**Results:**

Adoption was supported by teachers who valued sleep education and considered it a responsibility of schools. The intervention was generally well implemented, engaging, and aligned with teacher competencies and school structures. However, teachers questioned whether it would sufficiently improve adolescents' sleep health and questioned the intervention's optimal compatibility across educational tracks and grades. Adolescent satisfaction was moderate, showing differences among educational tracks.

**Implications for School Health Policy, Practice, and Equity:**

Involving stakeholders through participatory co‐creation is recommended to better address adolescents' diverse needs. Given the strong influence of the (social) environment on sleep, expanding Charge Your Brainzzz into a broader, systems‐oriented approach could yield greater and more lasting impact.

**Conclusions:**

This study provides insights into the adoption, implementation, and sustainment of Charge Your Brainzzz. While well received, further co‐creative tailoring and a broader systems approach are needed to improve its impact on sleep health.

**Trial Registration:**

ISRCTN36701918

## 1 Introduction

Poor sleep health is increasingly recognized as a public health issue [1] with serious implications for health and well‐being [2, 3, 4, 5]. Consequences include diminished mental health [6, 7], emotional and behavioral problems [8], an increased risk of overweight and obesity [9, 10], and it negatively impacts learning and school performance [11]. Adolescents are particularly vulnerable to developing poor sleep health [12, 13] due to numerous biological and (psycho)social factors [14, 15]. Teenagers in the Netherlands are no exception to this, as over half of adolescents aged 14–17 years sleep an hour less than the recommended 8–10 h per night [13], and nearly half of the adolescents do not wake up feeling rested [16]. Insufficient sleep duration, together with poor sleep quality, can result in daytime sleepiness, which is present in 18% of Dutch adolescents [13]. Moreover, a recent study found that one in three adolescents indicated symptoms of sleep reduction [17].

Given these worrisome developments in adolescent sleep health, effective health promotion programs are paramount. Since schools have the potential to reach large populations of adolescents at once, they have shown themselves to be a promising setting to promote healthy behavior [18, 19]. In the case of sleep health, unfortunately, results are still mixed [20, 21, 22]. While in some instances behavioral determinants such as knowledge were influenced, impacting actual sleep health and sleep‐related behaviors has proven to be complex and challenging [14, 15, 23, 24].

Considering the poor sleep health of today's adolescents in combination with the lack of school‐based interventions to address it, the Charge Your Brainzzz intervention was developed and evaluated on effectiveness [25]. It was shown to improve adolescents' sleep knowledge, attitude, and perceived behavioral control, but not other social‐cognitive determinants, sleep hygiene practices, or sleep outcomes [25]. As part of the evaluation study, a mixed methods process evaluation was conducted. To optimize the intervention's effectiveness, feasibility, appropriateness, and relevance, it is essential to better understand the (perceived) barriers and facilitators that affect its adoption, implementation, and sustainment [26]. Few school‐based sleep health interventions have been evaluated through a process evaluation [21]. Available results show that teachers view sleep education as important (i.e., a facilitator) while also experiencing a lack of time to implement it (i.e., a barrier) [21]. Given the lack of evaluations involving program users, this study aims to provide insights into the adoption, implementation, and sustainment processes of the Dutch school‐based intervention Charge Your Brainzzz from the perspectives of both teachers and adolescents.

## 2 Methods

A mixed‐methods process evaluation was conducted for the school‐based program “Charge Your Brainzzz” as part of a larger evaluation study [25]. The intervention was developed by the Dutch Brain Foundation Netherlands and Chrono@Work, neither of which were involved in the evaluation.

### 2.1 Participants and Recruitment

Secondary schools were recruited for the effect evaluation, which also included the current process evaluation for the intervention schools. Recruitment of schools took place through the Charge Your Brainzzz website, via the different participating local Public Health Services' (in Dutch: GGD) Healthy School Advisors, an online platform for school professionals, and via a magazine for biology teachers. This resulted in 10 participating schools, five of which were intervention schools that all participated in the process evaluation.

Teachers invited their second‐ and third‐grade students to take part in the study and complete questionnaires, including a satisfaction survey for the process evaluation. All teachers who delivered the intervention were approached for an interview as part of the current study. Written informed consent was obtained from teachers, adolescents, and parents.

Teachers were interviewed after the intervention, and adolescents completed a digital questionnaire at the follow‐up measurement of the trial (±3 months post‐intervention). Data collection occurred between September 2018 and January 2019.

### 2.2 Charge Your Brainzzz Intervention

The main aim of Charge Your Brainzzz is to stimulate adolescents' sleep health by targeting several social‐cognitive behavioral determinants such as knowledge and attitudes about sleep health, the biological clock, and sleep–wake rhythms. Throughout the intervention, adolescents also evaluate their own sleep habits and learn how to improve them by adopting healthy sleep hygiene practices. More details on the theoretical foundation of the intervention can be found elsewhere [25].

Charge Your Brainzzz targets students in the second or third grade of Dutch secondary schools, aged generally between 13 and 15 years old. It is delivered by schools' own teachers or mentor teachers (i.e., a teacher who supports a class of students throughout the school year), and is designed to fit into Dutch secondary schools' biology curriculum. To align with the educational tracks of technical prevocational secondary education (in Dutch: VMBO‐T), as well as senior general secondary education and pre‐university education (in Dutch: HAVO and VWO), two slightly customized versions were created. In short, Charge Your Brainzzz consists of three 45‐min classroom‐based education sessions delivered by teachers, including a manual to deliver the lessons. The sessions include interactive in‐class assignments and are complemented by an educational website (www.chargeyourbrainzzz.nl). Additionally, the intervention includes homework assignments involving a serious game and assignments for adolescents and their parents. An overview of the intervention can be found in Table 1.

**TABLE 1:** Overview Charge Your Brainzzz intervention.

Lesson 1 (45 min)	Lesson 2 (45 min)	Lesson 3 (45 min)
Introduction	Introduction and feedback homework assignment	Introduction and feedback homework assignment
Animation “Sleep problems and consequences”	Animation “Biological clock”	Animation “Sleep tips”
Card game “Sleep disturbances”	Card game “Q&A snake”	Card game “Sleep habits”
Round‐up Homework assignment: Playing “The Game”	Round‐up Homework assignment: Playing “The Game” and completing the “Chronotype” assignment (adolescents and their parents)	Round‐up: quiz in class

### 2.3 Theoretical Framework and Measurement Instruments

#### 2.3.1 Theoretical Framework

The process evaluation followed the framework of Fleuren et al. [27] which outlines the main stages of innovation processes: adoption, implementation, and sustainment. It also incorporated the process evaluation indicators of Saunders et al. [28], and previous implementation research of van Nassau et al. [29].

The adoption phase refers to the decision‐making process by schools or teachers to start using the intervention, considering its relevance, recruitment, and reach. The implementation phase focuses on executing the intervention as planned, assessing dose (i.e., how much of the intended intervention was delivered as planned), fidelity (i.e., how closely the intervention is implemented in line with its protocol), expected/perceived effectiveness (i.e., the perception on how well the intervention achieves its intended outcomes), and satisfaction with the intervention (i.e., users and implementers perceptions). Sustainment addresses long‐term maintenance, including necessary adaptations and the intervention's integration into regular practice or policy.

For each phase, the hindering and facilitating factors were explored. Following van Nassau et al. [29], Fleuren et al.'s [27] categories were adapted to the school context: sociopolitical factors (e.g., rules, regulations and societal beliefs), organizational factors (e.g., decision‐making processes in the school, available time), user factors (e.g., teacher skills and knowledge), and program factors (e.g., program compatibility and complexity). A topic guide for the semistructured interviews with teachers and a questionnaire for adolescents were developed based on this framework.

#### 2.3.2 Interviews With Teachers

The interviews with teachers aimed to gain insight into teachers' overall experience with the intervention and identify factors that potentially hampered or facilitated its adoption, implementation, and sustainment across various contextual areas of the school setting. In short, we explored to what extent teachers believed that schools have a responsibility to promote students' sleep health. Regarding the adoption process, we focused on how the decision was made to adopt Charge Your Brainzzz, whether the school first ensured broad support among staff, and whether staff were involved in shaping implementation and sustainment strategies. For implementation, the interviews addressed whether teachers felt sufficiently competent and supported in providing the Charge Your Brainzzz lessons and what factors hindered or facilitated implementation. We also discussed whether they implemented the program as intended, whether they were satisfied with it, and whether they believed it contributed to promoting healthy sleep habits among students. Finally, regarding sustainment, we asked whether the program aligned with the existing curriculum, what actions teachers believed were necessary to embed Charge Your Brainzzz structurally into the school curriculum, and whether they saw any barriers or opportunities for doing so. More details on the exact interview guideline can be found in Appendix A. Furthermore, demographic characteristics of teachers, including gender, years of work experience, and teacher type, were collected. One‐on‐one telephone interviews were conducted by a trained interviewer (M.‐B.M.R.I.), the principal researcher, who was not involved in developing the intervention.

#### 2.3.3 Questionnaire for Adolescents

A short questionnaire was administered to adolescents in the intervention schools to evaluate their satisfaction with Charge Your Brainzzz. It included an overall rating (i.e., 1 = very bad to 10 = excellent) of the lessons and the accompanying serious game, as well as ratings of specific aspects such as difficulty, the number of lessons, the homework assignment involving parents, the clarity of instructions, and program layout. Additionally, adolescents were asked to what extent the program motivated them to improve their sleep, with responses on a 5‐point Likert scale (including “not applicable” for those who did not attend the lessons, complete the homework assignments, or play the serious game). Finally, two open‐ended questions allowed for feedback on positive aspects and suggestions for improvement. Demographic questions included gender, educational track, and grade. The questionnaire can be found in Appendix B.

### 2.4 Data Analysis

Educational tracks were categorized into prevocational secondary education (i.e., VMBO‐T) and senior general/preuniversity education (i.e., HAVO, VWO, Gymnasium). Data were stratified by educational tracks to distinguish between the two program versions. *t* Tests were used to test for significant differences in mean scores. Data analysis was performed using SPSS version 27.

All interviews were recorded, transcribed verbatim, and analyzed in MAXQDA 2022. The principal investigator (M.‐B.M.R.I.) open‐coded the transcripts, which were then independently checked and additionally coded by a second researcher (M.M.V.S.). Using thematic analysis [30], three authors (M.‐B.M.R.I, M.M.V.S., F.V.N.) held a session to create a codebook, sorting codes by the adoption, implementation, and sustainment phases and grouping them into themes based on Fleuren et al.'s categories [27]: sociopolitical, organizational, user, and program context. Data were analyzed according to the codebook, and summaries were integrated into the results section. The most relevant and illustrative quotes were selected through team discussion.

## 3 Results

Interviews were conducted with 12 out of the 22 teachers (55%) who implemented the intervention. The interviews lasted 28 min on average (range 17–47 min). Additionally, a total of 372 adolescents (61% of the number of adolescents at baseline) completed the process evaluation questionnaire regarding their experiences with the intervention. Table 2 presents the characteristics of both the teachers and the adolescents.

**TABLE 2:** Characteristics of interviewed teachers and adolescents.

Teachers		Adolescents	
Total: *N* = 12	Number (%)	Total: *N* = 372	Number (%)
*Gender*		*Gender*	
Men	4 (33%)	Boys	151 (41%)
Women	8 (66%)	Girls	221 (59%)
Number of intervention schools	5	*Educational track*	
Work experience (years)	Mean 13.2 years (range: 1–36)	Prevocational secondary education	98 (26%)
		Senior general/preuniversity education	272 (74%)
*Teacher type* ^a^		*Grade*	
Biology	8	Second grade	209 (56%)
Mentor teacher	6	Third grade	163 (44%)

^a^ A teacher could be both a biology teacher and a mentor teacher.

### 3.1 Adoption

Five out of the 12 teachers were the initiator/project coordinator and were responsible for implementing Charge Your Brainzzz at their school. The remaining teachers indicated their support for the project and were invited to participate by the initiator/project coordinator at their school.

#### 3.1.1 Sleep Education Aligns With the Responsibility of Schools

Sleep health was not part of the curriculum in any school, despite teachers acknowledging its importance. This created an opportunity for an intervention like Charge Your Brainzzz. This was strengthened by all teachers indicating they perceived sleep education as a responsibility of school, being a place to naturally reach all adolescents. In addition, schools felt they had a broader societal role in health promotion, including sleep education alongside other topics such as smoking or sexual education. While teachers believed sleep education should primarily be the responsibility of parents, given schools' limited influence over home life, they recognized that not all parents can fulfill this role. As a result, teachers felt schools also had a role to play, including identifying sleep problems and providing a space for open discussions on important topics.

*Teacher: School is the designated place to address students' sleep behavior: that's where the burden is most felt*.

A facilitator mentioned flexibility within the curriculum, which was mostly the case when Charge Your Brainzzz was incorporated into the annual plan at the start of the year or when there was no fixed annual plan for the objectives, activities, and timelines of the academic year. Conversely, fixed annual plans and a full biology curriculum were mentioned as barriers.

#### 3.1.2 Teachers' Need to Address the Topic of Sleep

Teachers consistently considered sleep health important to address in school, especially since they increasingly experience the consequences of sleep‐deprived students in their classes. To engage less enthusiastic teachers, interviewees suggested emphasizing the importance of sleep education and involving them in the decision‐making process to foster collective responsibility for implementing Charge Your Brainzzz.

### 3.2 Implementation

#### 3.2.1 Preparation Work by Teachers

Teachers noted that the content of the intervention aligned well with the competencies of biology teachers and mentor teachers. While some teachers needed time to familiarize themselves with the intervention and its lessons, the majority found that the preparation time was comparable to that of their usual lessons.

#### 3.2.2 Dose and Implementation Fidelity

The majority of teachers delivered all three lessons, although some combined them into two sessions due to time constraints. Most teachers provided all work forms, although sometimes certain work forms were skipped due to time constraints. The homework assignment was given in most cases, with one teacher integrating the assignment into a test to encourage turning in the assignment. Four teachers added something to the lessons, such as an interactive quiz, a documentary clip, or a short sleep questionnaire to discuss in class.

*Teacher: But I also found that if you really wanted to do the assignments properly and discuss them, there was really too little time for that. So yes, we did not have more time than those lessons, so yeah…*

#### 3.2.3 Satisfaction

Overall, teachers were satisfied with the intervention, noting its engaging nature and inclusion of all elements necessary for enjoyable lessons. The majority was satisfied with the lesson content as it covered interesting theory about sleep and sleep hygiene practices. Also, all teachers mentioned the variety of work forms as one of the benefits of the intervention. However, the content and work forms of the program were not always suitable for all grades and educational tracks. These differences were not primarily related to the relevance of the sleep health theme or the level of engagement with it, but rather to comprehension, particularly regarding the language used. For example, teachers mentioned that for prevocational students, the content was sometimes too theoretical. Differences between second‐ and third‐grade classes, on the other hand, were more related to engagement with the intervention materials, with some work forms perceived as too childish by older students. Additionally, some teachers felt the serious game and some work forms did not allow enough reflection for students to relate the content personally. While most teachers agreed that three lessons were sufficient, some preferred two to save time and encourage broader participation from other teachers. Although the teacher manual and website provided adequate guidance for delivering the lessons, some teachers found them too extensive and challenging to navigate.

Table 3 presents the satisfaction with Charge Your Brainzzz as reported by adolescents in the survey. Overall, they were positive, although they enjoyed the lessons more (6.4 out of 10 score on average) than “The Game” (5.5 score on average). Teachers reported similar experiences of their students with the serious game. Additionally, there was a significant discrepancy in satisfaction with the intervention and the serious game between educational tracks. In general, adolescents in senior general and pre‐university education tracks provided higher overall ratings compared to those in prevocational educational tracks. The majority of all adolescents found the lessons easy to follow and believed that three lessons were enough for learning about sleep.

*Adolescent: It [Charge Your Brainzzz] helps you to understand why you sometimes have trouble sleeping and shows you how to improve your sleep*.

**TABLE 3:** Satisfaction with Charge Your Brainzzz (CYB) by adolescents.

	Prevocational secondary education (±*N* = 77), mean (SD)	Senior general/preuniversity education (±*N* = 213), mean (SD)
Charge Your Brainzzz—lessons (1–10 score)	6.3 (1.3)^a^	6.6 (1.5)^a^
Charge Your Brainzzz—serious game (1–10 score)	5.1 (1.5)^a^	5.9 (2.0)^a^
*Statements*	*(strongly) Agree*	
I liked the lessons	31.4%	44.8%
The lessons were easy to follow	67.5%	75.7%
Three lessons were sufficient to learn more about sleep	54.3%	67.1%
I liked The Game	19.0%	35.8%
I liked the homework assignment that I made with my parents	20.0%	17.0%
I liked the way CYB looked	35.9%	41.9%
I learned a lot from the lessons	35.9%	50.2%
CYB motivated me to sleep better	17.9%	26.1%

*Note*: Overall satisfaction was measured on a 10‐point rating scale: *very bad* (1) to *excellent* (10). Statements were measured on a 5‐point scale: *strongly disagree* (1) to *strongly agree* (5).

^a^ Significant difference between groups.

#### 3.2.4 Perceived Effectiveness

Teachers believed that the intervention provided valuable insights into adolescents' sleeping habits, encouraged critical reflection on their own sleep habits, increased their knowledge about sleep, and raised awareness. However, they were doubtful whether this would be enough to impact actual sleep behavior, especially in the long term. These perspectives align with adolescent data, where about one‐third (prevocational track) and half (senior general/preuniversity education) of the adolescents reported learning from the lessons, but only half of these groups indicated that the intervention motivated them to sleep better. According to teachers, adolescents often do not perceive their sleep as problematic or do not mind being sleepy at school. Another factor they felt hindered Charge Your Brainzzz's potential effects was that adolescents prioritize other things over sleeping well and that they face many distractions that impede the development of healthy sleeping habits. Some teachers suggested that the intervention might be more effective if the topic of sleep were revisited annually in each grade throughout the school years. This ongoing focus on sleep could potentially reinforce the importance of prioritizing healthy sleep among adolescents.

*Teacher: I think it [Charge Your Brainzzz] helped for a while. Students started enthusiastically, but it is mainly short‐term. I am very positive about the program, but it is really just for a little while*.

#### 3.2.5 Important, but Other Priorities

There was sufficient support among all schools for the implementation of the program, but sometimes the intervention did not receive sufficient priority in practice. For example, some teachers mentioned that while their colleagues acknowledged the importance of sleep education, the annual learning objectives, that is, student cognitive development, were perceived as more important, which resulted in sleep education being given a lower priority. A barrier for implementation arose when one teacher (*N* = 1) was solely responsible for implementing the intervention, highlighting the need for more broadly shared responsibility.

### 3.3 Sustainment

#### 3.3.1 Distribute Lessons Over the School Year

All teachers wanted to continue the intervention in the following year; although the majority indicated they would make some adjustments to the lessons or combine three lessons into two. Especially the mentor teachers preferred to distribute the lessons throughout the year, allowing for the inclusion of other topics or tasks during mentor hours.

#### 3.3.2 Incorporation Into the Overall Learning Objectives

Identified barriers to the sustainment of Charge Your Brainzzz included an overloaded curriculum, the dependency of project continuation on individual teachers' motivation, and the need for an initiator who takes the responsibility. To facilitate the structural integration of sleep education in schools, factors such as incorporating sleep as a subject into the school's overall learning objectives and incorporating it as standard teaching material in biology textbooks were suggested.

#### 3.3.3 Alignment of the Program With Biology and Mentor Hours

The majority of teachers felt that the intervention was better suited for courses other than biology, such as social studies, philosophy, or mentor hours. Sleep is a topic that warrants class discussions, with mentor hours offering space for such interactions, facilitated by a teacher whom students are better acquainted with. It was also noted that fostering an open and safe atmosphere is essential for effectively discussing sleeping habits. This shows that teachers are aware of the sensitivity of the topic of sleep, as it involves personal experiences, beliefs, habits, and stories of adolescents.

*Teacher: The theory of sleep can be covered in biology, but beyond the theory, it also involves the effects on, for example, school performance, making it much more suitable for a mentor hour in terms of content. Just like [themes as] bullying or alcohol use*.

## 4 Discussion

This study consisted of a comprehensive process evaluation to determine the factors influencing the adoption, implementation, and sustainment of the Charge Your Brainzzz intervention, a program to promote sleep health among adolescents in Dutch secondary schools.

The adoption of Charge Your Brainzzz was facilitated by all teachers recognizing the importance of addressing sleep health among their students, since teachers often have to deal with its negative consequences at school. Beyond their primary role as educators, teachers perceived sleep education as part of schools' broader societal responsibility. Despite this, sleep health is not yet an integrated part of the Dutch secondary school curriculum. This provided an opportunity for Charge Your Brainzzz, as the first sleep health‐promoting intervention available. However, an overloaded curriculum, a common barrier to school‐based health interventions [29, 31], remained a challenge. Despite this, it did not hinder its uptake and implementation in intervention schools in the current study. The limited flexibility of Dutch secondary schools' curricula to integrate programs such as Charge Your Brainzzz underscores the importance of aligning such interventions with existing school structures and goals.

Regarding implementation, teachers were satisfied with the intervention, found it engaging, and it aligned well with their competencies. This is important, as sleep education is most effective when delivered by students' regular teachers [32]. However, teachers did express concerns about the intervention's compatibility with different educational tracks and grades, potentially hindering its implementation. This concern was echoed in adolescents' feedback, with those in prevocational tracks showing less appreciation for the intervention compared to students in the other tracks, mostly because they found the materials too theoretical, which made the information harder to understand. Additionally, the serious game component of Charge Your Brainzzz received little enthusiasm, particularly from adolescents in the prevocational tracks who were more critical. Therefore, greater attention to tailoring sleep health interventions to different educational tracks and learning styles is necessary [21].

## 5 Implications for School Health Policy, Practice, and Equity

The results of this study demonstrated that Charge Your Brainzzz is a promising school‐based intervention to address sleep health. The intervention is cost‐free, with content and a practical implementation manual for teachers readily available for schools. Teachers indicated that they considered it an important theme and that sleep education aligns with the responsibility of schools and the skills of teachers. However, to better promote equity and to improve students' satisfaction, the intervention should be better tailored to suit the needs of students in different educational tracks. This requires active involvement of all stakeholders that receive or implement the intervention, including adolescents from all educational tracks, teachers, school boards, and health promotion professionals in both the design and implementation process. Such a participatory approach not only ensures the intervention's appropriateness but also facilitates integration into existing school infrastructures. Unfortunately, most school‐based health interventions, including those focused on sleep [20, 21], are developed with minimal input from the target population. For the development of Charge Your Brainzzz, teachers were consulted about the general outlines of the intervention, while adolescents provided their preferences regarding work forms, the layout of the serious game, the content, and its alignment with their knowledge level. However, neither group was actively involved in the actual development process. This may explain why student satisfaction with the program was only moderate; the intervention may not have fully reflected adolescents' needs, preferences, and lived experiences. Given adolescents' drive for autonomy [33], greater involvement of the target group could foster a stronger sense of ownership, leading to increased engagement and appreciation. In this light, adopting a more participatory or user‐centered approach in the future development of the intervention is essential, as it is key to enhancing the program's relevance, acceptability, implementation, and ultimately its effectiveness [34].

Although teachers believed that Charge Your Brainzzz raised adolescents' awareness of sleep health, they did not expect it to result in significant, sustained changes in sleep health. This poses a challenge for the sustainment of the intervention. This is reflected in the effect evaluation: while adolescents showed improved knowledge, attitudes, and perceived behavioral control, their sleep hygiene and sleep outcomes remained unchanged [25]. Teachers noted that various factors beyond adolescents' social‐cognitive determinants, which are currently targeted in the intervention, impede their sleep habits. Studies on sleep and health interventions suggest school‐based efforts alone are necessary but insufficient for durable behavior change [21, 22, 35]. Environmental factors such as peer influences, online and smartphone habits, family dynamics, along with school policies such as early starting times, significantly impact adolescent sleep, too [14, 15, 36, 37, 38, 39]. Therefore, while Charge Your Brainzzz shows promise for sleep health education, evolving it into a more integrated approach that encompasses the school, home, online, and peer environments seems necessary.

When implementing school‐based interventions, viewing schools as complex adaptive systems can be valuable [40]. This perspective recognizes schools as dynamic, interconnected systems where components interact unpredictably, adapting to changes and shaping outcomes. Successful implementation, such as implementing a program like Charge Your Brainzzz, may require shifts in core beliefs. For instance, since schools prioritize education and cognitive development, framing sleep health as vital for learning and socialization might be important. This approach aligns with our findings, where teachers were eager to integrate Charge Your Brainzzz, but concerned it would not be prioritized in an already crowded and inflexible curriculum. These concerns reflect previous studies showing that time constraints and additional burden on the curriculum were significant concerns [21, 23, 32]. This indicates that teachers primarily view schools as places for cognitive learning, prioritizing academic education over health education, with sleep health still seen as part of the latter. Therefore, redefining schools as environments supporting holistic development, beyond just cognitive growth, could improve the implementation and sustainability of interventions like Charge Your Brainzzz.

## 6 Strengths and Limitations

A key strength of this study is its mixed‐methods design, combining qualitative and quantitative measures from both teachers and adolescents to provide a more in‐depth evaluation of Charge Your Brainzzz. Another strength is the comprehensiveness of the evaluation, providing a thorough and detailed understanding of the implementation of the program. Additionally, data saturation was achieved during interviews, indicating that key topics were thoroughly addressed.

A limitation was that the questionnaire for adolescents was conducted approximately 3 months after the intervention, potentially reducing participation and introducing recall bias. Administering it post‐intervention could have addressed this. Additionally, due to time constraints, adolescents were not interviewed, limiting the ability to gather more in‐depth insights.

## 7 Conclusion

This study provides insights into the factors influencing the adoption, implementation, and sustainment of the Charge Your Brainzzz adolescent sleep health intervention. The intervention was generally well‐received, well‐implemented, and moderately appreciated by its target population. To enhance its uptake, implementation, and sustainment, it should be better tailored to its target population. This requires the close involvement of adolescents, teachers, and other implementers throughout the intervention (re)development process through participatory co‐creation processes. Additionally, to increase its impact on sleep health, further development of Charge Your Brainzzz into a broader, integrated approach with a systems science focus seems necessary to create more impactful changes in adolescent sleep health.

## Ethics Statement

The Medical Ethical Committee of the Amsterdam UMC (VUmc) approved this study (reference number 2018.248).

## Conflicts of Interest

The authors declare no conflicts of interest.

## Data Availability

The data that support the findings of this study are available from the corresponding author upon reasonable request.

## Appendix A Topic Guide Interviews Teachers

### Informed Consent Information

Do you consent to the audio recording of this interview? The recording will be handled confidentially. It will be transcribed and anonymized, ensuring that the transcript cannot be traced back to your personal information. If at any point you wish to stop the interview or take a break, you are free to do so. Do you have any questions before we begin?

### Purpose of the Research and This Interview

The purpose of this interview is to gain insight into your experience and opinion of the Charge Your Brainzzz lesson package, as well as its implementation at your school in both the short and long term. Your feedback will help shape the content and format of the lesson package for future improvements.

Please note that this interview is focused solely on the lesson package, involving the three lessons and The Game. This interview is not about organizing and conducting the three research measurements. We aim to get a comprehensive view, so we are interested in hearing both what went well and what could be improved.

### Introduction

- Could you briefly introduce yourself?
  ○ Your name, background, and how long you have been in the profession
- To what extent do you believe it is important to focus on sleep education in schools?
  ○ To what extent do you think schools should be the place to promote healthy sleep habits among students?
  ○ To what extent do you believe it is your school's responsibility to encourage healthy sleep patterns among its students?
  ○ How important do you think it is for your school to present itself as one that values student health?
- How important do you think your colleagues consider addressing the topic of sleep at school?
- How significant do you think parents and students find the focus on sleep education at school?

### Decision‐Making Process

- Who among you was involved in the decision‐making process to participate in Charge Your Brainzzz?
  ○ If so, in what way?
  ○ What were your thoughts on this involvement?
  ○ What, if anything, would you have liked to see done differently; and why? How has this influenced the implementation of Charge Your Brainzzz at your school?
- What motivated you or your school to begin working with Charge Your Brainzzz?

### Implementation of the Intervention

- To what extent was there support among your fellow teachers for introducing the lesson package?
- How many teachers at your school have delivered the Charge Your Brainzzz lessons?
- What was the response from parents and students regarding the introduction of the lesson package?
- What types of support or information were available to assist you in teaching the lessons?
  ○ Was this support sufficient? If not, what additional support would have been ideal?
- Did you feel adequately competent to use this lesson package?
- What factors have facilitated or could facilitate the implementation of Charge Your Brainzzz?
  ○ Are there sufficient hours and teachers available for implementing Charge Your Brainzzz?
  ○ Does your school have the necessary resources to implement and execute Charge Your Brainzzz? (e.g., projectors, computers, printing capabilities for assignments)? If not, what is lacking?
- Are there any other programs/interventions at your school that address sleep education?
- Does Charge Your Brainzzz integrate well with the regular curriculum? If so, in which subject area (e.g., biology)?
- In this research, we actively introduced Charge Your Brainzzz to your attention. What would be an effective way or channel to normally introduce a lesson package like this to schools/teachers?

### Lesson Execution and Feedback on Materials

- What is your overall opinion of Charge Your Brainzzz?
  ○ What are its strengths?
  ○ What are its areas for improvement?
- What do you think the students' overall opinion of Charge Your Brainzzz is?
- To what extent was the lesson package implemented as intended?
  ○ Were all three lessons taught?
  ○ Were all teaching methods used? (i.e., one animation per lesson; card game; Q&A snake; quiz; sleep habit cards)
  ○ Were all homework assignments given? (i.e., chronotype assignment for parents and students; playing The Game)
- To what extent did you adhere to the content of the lesson package as described in the teacher's guide?
- Did you make any adjustments to the lessons or their content? If so, why; and what changes were made?
- Over what time frame were the lessons delivered? (e.g., all in one week or spread over a longer period?)
  ○ Why was this time frame chosen? What worked well? What would you recommend?
- What do you think of the following aspects of Charge Your Brainzzz?
  ○ The materials.
  ○ The teaching methods.
  ○ The time investment required.
  ○ The number of lessons.
  ○ The target audience of the lesson package (e.g., should parents be more involved).
- What do you think the students think of:
  ○ The materials.
  ○ The teaching methods.
  ○ The number of lessons.

### Recruitment

- Is it necessary to receive additional information or an introduction to the lesson package before starting Charge Your Brainzzz? If so, what form should this take?

### Embedding in the School

- What is your opinion on incorporating Charge Your Brainzzz into your school's curriculum?
- If a new school principal or head of the biology department were to join your school, do you think there would be enough support within the school team to continue with Charge Your Brainzzz and integrate it into the curriculum?
  ○ Why or why not?
  ○ What resources or tools would be helpful for successfully embedding Charge Your Brainzzz at your school?
- What factors, in your opinion, facilitate or hinder the long‐term or structural implementation of Charge Your Brainzzz?

### Final Question

- To what extent do you think this lesson package and its associated materials contribute to promoting healthy sleep habits among students?

### Conclusion

- Are there any topics that were not discussed or were overlooked that you would like to address?

## Appendix B Questionnaire Adolescents

How would you rate the Charge Your Brainzzz lessons overall?

Very bad 1 2 3 4 5 6 7 8 9 10 Excellent

How would you rate The Game (the online game) overall?

Very bad 1 2 3 4 5 6 7 8 9 10 Excellent

Please indicate how much you agree with the statements below. If you did not participate in the lessons, complete the homework, or play The Game, please select “not applicable.”

**Table:**

	Totally disagree	Disagree	Neither agree nor disagree	Agree	Totally agree	Not applicable
I enjoyed the Charge Your Brainzzz lessons	☐	☐	☐	☐	☐	☐
I enjoyed playing The Game	☐	☐	☐	☐	☐	☐
I found the Charge Your Brainzzz lessons easy to follow	☐	☐	☐	☐	☐	☐
I found that three Charge Your Brainzzz lessons were sufficient to learn more about healthy sleep	☐	☐	☐	☐	☐	☐
I enjoyed completing the homework assignment about chronotypes with my parent(s)	☐	☐	☐	☐	☐	☐
I learned a lot from Charge Your Brainzzz	☐	☐	☐	☐	☐	☐
I liked the lay‐out of the Charge Your Brainzzz materials (for example: the quartet and the Q&A snake)	☐	☐	☐	☐	☐	☐
Charge Your Brainzzz has motivated me to improve my sleep	☐	☐	☐	☐	☐	☐

What was good about Charge Your Brainzzz? Please write down everything that comes to mind.

What could be improved about Charge Your Brainzzz? Please write down everything that comes to mind.

## References

[1] F. Louzada , “Adolescent Sleep: A Major Public Health Issue,” Sleep Science 12, no. 1 (2019): 1. 31105887 10.5935/1984-0063.20190047 PMC6508948

[2] T. Shochat , M. Cohen‐Zion , and O. Tzischinsky , “Functional Consequences of Inadequate Sleep in Adolescents: A Systematic Review,” Sleep Medicine Reviews 18, no. 1 (2014): 75–87. 23806891 10.1016/j.smrv.2013.03.005

[3] J. Owens and Adolescent Sleep Working Group; Committee on Adolescence , “Insufficient Sleep in Adolescents and Young Adults: An Update on Causes and Consequences,” Pediatrics 134, no. 3 (2014): e921–e932. 25157012 10.1542/peds.2014-1696 PMC8194472

[4] L. Matricciani , C. Paquet , B. Galland , M. Short , and T. Olds , “Children's Sleep and Health: A Meta‐Review,” Sleep Medicine Reviews 46 (2019): 136–150. 31121414 10.1016/j.smrv.2019.04.011

[5] J.‐P. Chaput , C. E. Gray , V. J. Poitras , et al., “Systematic Review of the Relationships Between Sleep Duration and Health Indicators in School‐Aged Children and Youth,” Applied Physiology, Nutrition, and Metabolism 41, no. 6 (2016): S266–S282. 10.1139/apnm-2015-0627 27306433

[6] T. J. Saunders , C. E. Gray , V. J. Poitras , et al., “Combinations of Physical Activity, Sedentary Behaviour and Sleep: Relationships With Health Indicators in School‐Aged Children and Youth,” Applied Physiology, Nutrition, and Metabolism 41, no. 6 (2016): S283–S293. 10.1139/apnm-2015-0626 27306434

[7] S. Uccella , R. Cordani , F. Salfi , et al., “Sleep Deprivation and Insomnia in Adolescence: Implications for Mental Health,” Brain Sciences 13, no. 4 (2023): 569. 37190534 10.3390/brainsci13040569 PMC10136689

[8] C. A. Palmer and C. A. Alfano , “Sleep and Emotion Regulation: An Organizing, Integrative Review,” Sleep Medicine Reviews 31 (2017): 6–16. 26899742 10.1016/j.smrv.2015.12.006

[9] Y. Fatima , S. Doi , and A. Mamun , “Longitudinal Impact of Sleep on Overweight and Obesity in Children and Adolescents: A Systematic Review and Bias‐Adjusted Meta‐Analysis,” Obesity Reviews 16, no. 2 (2015): 137–149. 25589359 10.1111/obr.12245

[10] M. A. Miller , M. Kruisbrink , J. Wallace , C. Ji , and F. P. Cappuccio , “Sleep Duration and Incidence of Obesity in Infants, Children, and Adolescents: A Systematic Review and Meta‐Analysis of Prospective Studies,” Sleep 41, no. 4 (2018): zsy018. 10.1093/sleep/zsy018 29401314

[11] J. F. Dewald , A. M. Meijer , F. J. Oort , G. A. Kerkhof , and S. M. Bögels , “The Influence of Sleep Quality, Sleep Duration and Sleepiness on School Performance in Children and Adolescents: A Meta‐Analytic Review,” Sleep Medicine Reviews 14, no. 3 (2010): 179–189. 20093054 10.1016/j.smrv.2009.10.004

[12] G. Gariepy , S. Danna , I. Gobiņa , et al., “How Are Adolescents Sleeping? Adolescent Sleep Patterns and Sociodemographic Differences in 24 European and North American Countries,” Journal of Adolescent Health 66, no. 6 (2020): S81–S88. 10.1016/j.jadohealth.2020.03.013 32446613

[13] D. Kocevska , T. S. Lysen , A. Dotinga , et al., “Sleep Characteristics Across the Lifespan in 1.1 Million People From The Netherlands, United Kingdom and United States: A Systematic Review and Meta‐Analysis,” Nature Human Behaviour 5, no. 1 (2021): 113–122. 10.1038/s41562-020-00965-x 33199855

[14] S. J. Crowley , A. R. Wolfson , L. Tarokh , and M. A. Carskadon , “An Update on Adolescent Sleep: New Evidence Informing the Perfect Storm Model,” Journal of Adolescence 67 (2018): 55–65. 29908393 10.1016/j.adolescence.2018.06.001 PMC6054480

[15] D. M. Heemskerk , V. Busch , J. T. Piotrowski , W. E. Waterlander , C. M. Renders , and M. M. van Stralen , “A System Dynamics Approach to Understand Dutch Adolescents' Sleep Health Using a Causal Loop Diagram,” International Journal of Behavioral Nutrition and Physical Activity 21, no. 1 (2024): 34. 38519989 10.1186/s12966-024-01571-0 PMC10958857

[16] S. Leone , A. Van der Poel , K. Beers , L. Rigter , E. Zantinge , and M. Savelkoul , Slechte slaap: een probleem voor de volksgezondheid (Trimbos Instituut, Netherlands Institute of Mental Health and Addiction, 2018).

[17] M. P. Glasbeek , M.‐B. M. R. Inhulsen , V. Busch , and M. M. van Stralen , “Sleep Reduction in Adolescents: Socio‐Demographic Factors and the Mediating Role of Sleep Hygiene Practices,” Sleep Epidemiology 2 (2022): 100024.

[18] S. Kriemler , U. Meyer , E. Martin , E. M. van Sluijs , L. B. Andersen , and B. W. Martin , “Effect of School‐Based Interventions on Physical Activity and Fitness in Children and Adolescents: A Review of Reviews and Systematic Update,” British Journal of Sports Medicine 45, no. 11 (2011): 923–930. 21836176 10.1136/bjsports-2011-090186 PMC3841814

[19] I. De Bourdeaudhuij , E. Van Cauwenberghe , H. Spittaels , et al., “School‐Based Interventions Promoting Both Physical Activity and Healthy Eating in Europe: A Systematic Review Within the HOPE Project,” Obesity Reviews 12, no. 3 (2011): 205–216. 20122137 10.1111/j.1467-789X.2009.00711.x

[20] R. Gruber , “School‐Based Sleep Education Programs: A Knowledge‐To‐Action Perspective Regarding Barriers, Proposed Solutions, and Future Directions,” Sleep Medicine Reviews 36 (2017): 13–28. 27818085 10.1016/j.smrv.2016.10.001

[21] S. Blunden and G. Rigney , “Lessons Learned From Sleep Education in Schools: A Review of Dos and Don'ts,” Journal of Clinical Sleep Medicine 11, no. 6 (2015): 671–680. 25766709 10.5664/jcsm.4782 PMC4442228

[22] J. Cassoff , B. Knäuper , S. Michaelsen , and R. Gruber , “School‐Based Sleep Promotion Programs: Effectiveness, Feasibility and Insights for Future Research,” Sleep Medicine Reviews 17, no. 3 (2013): 207–214. 23063417 10.1016/j.smrv.2012.07.001

[23] S. L. Blunden , J. Chapman , and G. A. Rigney , “Are Sleep Education Programs Successful? The Case for Improved and Consistent Research Efforts,” Sleep Medicine Reviews 16, no. 4 (2012): 355–370. 22104441 10.1016/j.smrv.2011.08.002

[24] K. F. Chung , M. S. Chan , Y. Y. Lam , C. S. Y. Lai , and W. F. Yeung , “School‐Based Sleep Education Programs for Short Sleep Duration in Adolescents: A Systematic Review and Meta‐Analysis,” Journal of School Health 87, no. 6 (2017): 401–408. 28463450 10.1111/josh.12509

[25] M.‐B. M. R. Inhulsen , V. Busch , and M. M. van Stralen , “Effect Evaluation of a School‐Based Intervention Promoting Sleep in Adolescents: A Cluster‐Randomized Controlled Trial,” Journal of School Health 92, no. 6 (2022): 550–560. 35315076 10.1111/josh.13175 PMC9314837

[26] J. A. Durlak and E. P. DuPre , “Implementation Matters: A Review of Research on the Influence of Implementation on Program Outcomes and the Factors Affecting Implementation,” American Journal of Community Psychology 41 (2008): 327–350. 18322790 10.1007/s10464-008-9165-0

[27] M. Fleuren , K. Wiefferink , and T. Paulussen , “Determinants of Innovation Within Health Care Organizations: Literature Review and Delphi Study,” International Journal for Quality in Health Care 16, no. 2 (2004): 107–123. 15051705 10.1093/intqhc/mzh030

[28] R. P. Saunders , M. H. Evans , and P. Joshi , “Developing a Process‐Evaluation Plan for Assessing Health Promotion Program Implementation: A How‐To Guide,” Health Promotion Practice 6, no. 2 (2005): 134–147. 15855283 10.1177/1524839904273387

[29] F. van Nassau , A. S. Singh , D. Broekhuizen , W. van Mechelen , J. Brug , and M. J. Chinapaw , “Barriers and Facilitators to the Nationwide Dissemination of the Dutch School‐Based Obesity Prevention Programme DOiT,” European Journal of Public Health 26, no. 4 (2016): 611–616. 26873859 10.1093/eurpub/ckv251

[30] A. Castleberry and A. Nolen , “Thematic Analysis of Qualitative Research Data: Is It as Easy as It Sounds?,” Currents in Pharmacy Teaching and Learning 10, no. 6 (2018): 807–815. 30025784 10.1016/j.cptl.2018.03.019

[31] S. Cassar , J. Salmon , A. Timperio , et al., “Adoption, Implementation and Sustainability of School‐Based Physical Activity and Sedentary Behaviour Interventions in Real‐World Settings: A Systematic Review,” International Journal of Behavioral Nutrition and Physical Activity 16 (2019): 1–13. 31791341 10.1186/s12966-019-0876-4 PMC6889569

[32] G. Rigney , A. Watson , J. Gazmararian , and S. Blunden , “Update on School‐Based Sleep Education Programs: How Far Have We Come and What Has Australia Contributed to the Field?,” Sleep Medicine 80 (2021): 134–157. 33607553 10.1016/j.sleep.2021.01.061

[33] H. J. Spear and P. Kulbok , “Autonomy and Adolescence: A Concept Analysis,” Public Health Nursing 21, no. 2 (2004): 144–152. 14987214 10.1111/j.0737-1209.2004.021208.x

[34] Y. Anyon , K. Bender , H. Kennedy , and J. Dechants , “A Systematic Review of Youth Participatory Action Research (YPAR) in the United States: Methodologies, Youth Outcomes, and Future Directions,” Health Education & Behavior 45, no. 6 (2018): 865–878. 29749267 10.1177/1090198118769357

[35] W. E. Waterlander , A. Luna Pinzon , A. Verhoeff , et al., “A System Dynamics and Participatory Action Research Approach to Promote Healthy Living and a Healthy Weight Among 10–14‐Year‐Old Adolescents in Amsterdam: The LIKE Programme,” International Journal of Environmental Research and Public Health 17, no. 14 (2020): 4928. 32650571 10.3390/ijerph17144928 PMC7400640

[36] K. A. Bartel , M. Gradisar , and P. Williamson , “Protective and Risk Factors for Adolescent Sleep: A Meta‐Analytic Review,” Sleep Medicine Reviews 21 (2015): 72–85. 25444442 10.1016/j.smrv.2014.08.002

[37] M. A. Short , M. Gradisar , H. Wright , L. C. Lack , H. Dohnt , and M. A. Carskadon , “Time for Bed: Parent‐Set Bedtimes Associated With Improved Sleep and Daytime Functioning in Adolescents,” Sleep 34, no. 6 (2011): 797–800. 21629368 10.5665/SLEEP.1052 PMC3098947

[38] J. M. Bowers and A. Moyer , “Effects of School Start Time on Students' Sleep Duration, Daytime Sleepiness, and Attendance: A Meta‐Analysis,” Sleep Health 3, no. 6 (2017): 423–431. 29157635 10.1016/j.sleh.2017.08.004

[39] A. G. Wheaton , D. P. Chapman , and J. B. Croft , “School Start Times, Sleep, Behavioral, Health, and Academic Outcomes: A Review of the Literature,” Journal of School Health 86, no. 5 (2016): 363–381. 27040474 10.1111/josh.12388 PMC4824552

[40] S. R. Rosas , “Systems Thinking and Complexity: Considerations for Health Promoting Schools,” Health Promotion International 32, no. 2 (2017): 301–311. 26620709 10.1093/heapro/dav109

